# Antibody-independent capture of circulating tumor cells of non-epithelial origin with the ApoStream^®^ system

**DOI:** 10.1371/journal.pone.0175414

**Published:** 2017-04-12

**Authors:** Priya Balasubramanian, Robert J. Kinders, Shivaani Kummar, Vishal Gupta, David Hasegawa, Anoop Menachery, Scott M. Lawrence, Lihua Wang, Katherine Ferry-Galow, Darren Davis, Ralph E. Parchment, Joseph E. Tomaszewski, James H. Doroshow

**Affiliations:** 1 Laboratory of Human Toxicology and Pharmacology, Applied/ Developmental Research Support Directorate, Leidos Biomedical Research, Inc., Frederick National Laboratory for Cancer Research, Frederick, Maryland, United States of America; 2 Division of Cancer Treatment and Diagnosis, National Cancer Institute, Bethesda, Maryland, United States of America; 3 ApoCell, Inc., Houston, Texas, United States of America; University of Pécs Medical School, HUNGARY

## Abstract

Circulating tumor cells (CTCs) are increasingly employed for research and clinical monitoring of cancer, though most current methods do not permit the isolation of non-epithelial tumor cells. Furthermore, CTCs isolated with antibody-dependent methods are not suitable for downstream experimental uses, including *in vitro* culturing and implantation *in vivo*. In the present study, we describe the development, validation, and transfer across laboratories of a new antibody-independent device for the enrichment of CTCs from blood samples of patients with various cancer diagnoses. The ApoStream^®^ device uses dielectrophoresis (DEP) field-flow assist to separate non-hematopoietic cells from the peripheral blood mononuclear fraction by exposing cells in a laminar flow stream to a critical alternating current frequency. The ApoStream^®^ device was calibrated and validated in a formal cross-laboratory protocol using 3 different cancer cell lines spanning a range of distinct phenotypes (A549, MDA-MB-231, and ASPS-1). In spike-recovery experiments, cancer cell recovery efficiencies appeared independent of spiking level and averaged between 68% and 55%, depending on the cell line. No inter-run carryover was detected in control samples. Moreover, the clinical-readiness of the device in the context of non-epithelial cancers was evaluated with blood specimens from fifteen patients with metastatic sarcoma. The ApoStream^®^ device successfully isolated CTCs from all patients with sarcomas examined, and the phenotypic heterogeneity of the enriched cells was demonstrated by fluorescence in situ hybridization or with multiplex immunophenotyping panels. Therefore, the ApoStream^®^ technology expands the clinical utility of CTC evaluation to mesenchymal cancers.

## Introduction

The isolation and characterization of circulating tumor cells (CTCs) is of considerable research and clinical interest; however, most available methods rely on antibodies against epithelial cancer markers [1–3]. For patients with mesenchymal malignancies (e.g., sarcoma, and carcinoma undergoing epithelial-mesenchymal transition, or EMT), alternative CTC isolation platforms are needed. To address this unmet need, the National Cancer Institute (NCI) initiated a technology development project funded by the American Recovery and Reinvestment Act to develop an antibody-independent, clinically-suitable CTC isolation device for the capture of rare tumor cells from any cancer type. The developed technology was required to isolate viable CTCs of sufficient quality and purity to enable the analysis of molecular biomarkers of pharmacodynamic response to drug therapy. The technology also needed to be scalable for use on neonatal and small animal samples (volumes 0.1 to 1.0 mL), and CTC analysis had to be performed on the same aliquot of blood used for CTC enumeration. Finally, the instrument design had to have an open architecture, i.e., be amenable to the development of research assays by qualified clinical technicians.

Here, we report on the development and cross-laboratory validation of the ApoStream^®^ (ApoCell, Houston, TX) system, an antibody-independent methodology for isolating viable CTCs from epithelial and non-epithelial malignancies by exploiting the morphological and biophysical differences between malignant and normal blood cells [4]. Indeed, Gupta *et al*. reported >97% viability for MDA-MB-231 cells enriched with ApoStream^®^, and exponential growth comparable to control cells not processed with ApoStream^®^ [4]. The system uses dielectrophoresis (DEP) field-flow assist principles to separate cells with distinct biophysical properties, such as membrane capacitance, morphology, size, and electrical conductivity [5, 6]. Three solid tumor cell lines were used to demonstrate that the ApoStream^®^ technology works on a range of cancer types independent of expression levels of Epithelial Cell Adhesion Molecule (EpCAM), a biomarker commonly used in other isolation technologies for the analysis of CTCs originating from carcinomas. With this device, we also successfully isolated cancer cells from the blood of patients with Alveolar Soft Part Sarcoma (ASPS) and other soft tissue neoplasms.

## Materials and methods

### Ethics statement

The National Cancer Institute Institutional Review Board (NCI-IRB) approved the study. Written informed consent was obtained from all patients and healthy blood donors. Study design and conduct followed all applicable regulations, guidances, and local policies.

### Cell preparation

A549 lung cancer cells, MDA-MB-231 triple-negative breast cancer cells, and ASPS-1 sarcoma [7] cancer cells were used for spike-recovery experiments. A549 and MDA-MB-231 cell lines were purchased from ATCC (Manassas, VA); both testing sites used cell lines from the same lots. The identity of all cell lines was confirmed by Identifiler^®^ in the NCI Biological Testing Branch at the Frederick National Laboratory for Cancer Research (FNLCR; Frederick, MD). The ASPS-1 cell line was developed at the Frederick National Laboratory for Cancer Research [7], and was provided by Dr. Melinda Hollingshead of the Biological Testing Branch. A549 and MDA-MB-231 cells were cultured in RPMI medium (Lonza, Walkersville, MD), and ASPS-1 cells were cultured in DMEM:F12 medium (Lonza, Walkersville, MD). Culture medium was supplemented with 10% fetal bovine serum and 1% penicillin/streptomycin with regular media changes. Culture flasks were maintained at 37°C with 5% CO_2_ and 100% humidity. Cell quality was controlled and only cell cultures between 3–8 passages, 50–80% confluence, and >95% viability were used for the experiments. Cells were harvested from the same batch and cultured under similar conditions at both sites to minimize cell quality variance in controlled spiking experiments. Cells at pre-specified confluence were harvested and washed with growth medium prior to pre-labeling with a fluorescent cellular dye. Cells suspended in growth medium were pre-stained with 50 μM CellTracker™ Green (Invitrogen, Carlsbad, CA) for 30 min at 37°C, and washed twice in growth medium thereafter.

### Sample processing

**Fig 1:**
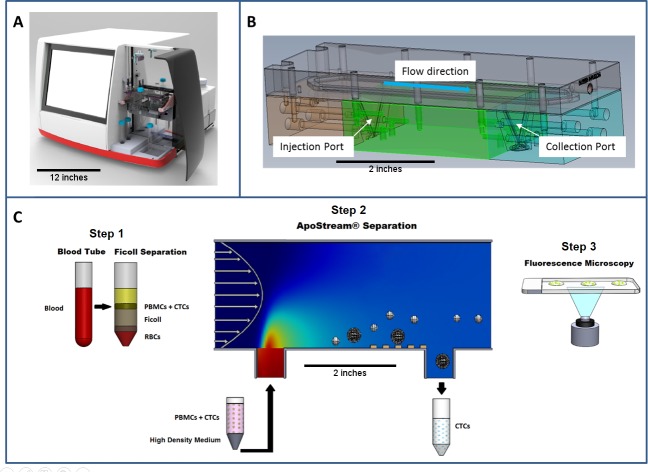
Schematic representations of the ApoStream^®^ device. **(A)** ApoStream^®^ prototype instrument. The device has a width of 24 inches, and a height of 17 inches. The scale bar represents 12 inches. **(B)** 3D CAD model of the flow chamber showing V-shaped injection and collection ports. The chamber measures 6 inches (length), by 2 inches (height), by 2 inches (depth). The scale bar represents 2 inches. **(C)** Step 1: Sample processing by Ficoll density gradient separation to isolate PBMCs and CTCs. Step2: DEP enrichment starting with sample injection, ion diffusion, DEP separation of CTCs from PBMCs, and CTC collection. The scale bar represents 2 inches. Step 3: downstream analysis using immunofluorescence or other techniques for CTC identification and enumeration.

The research-use-only (RUO) ApoStream^®^ (ApoCell, Houston, TX) instrument (Fig 1A and 1B) has been described in detail previously [4]. The flow chamber was disinfected and primed with absolute ethanol prior to each run, then preconditioned with a proprietary sucrose-based, low conductivity buffer. Ficoll-separated peripheral blood mononuclear cells (PBMCs) and cancer cells were suspended in a proprietary sample buffer optimized [4] for high recovery, density, and essential nutrients to maintain long-term cell viability, and loaded into the sample injection container (Fig 1C). Cells were introduced into the main flow channel at a flow rate of 35 μL/min using a syringe pump. Low conductivity eluate buffer was pumped through the main flow channel at a flow rate of 2.4 mL/min, resulting in a parabolic laminar flow stream across the ~ 400 μm height of the channel. Eluate and sample buffer flow rates were optimized to ensure that the levitation height of injected cells could be controlled with the DEP field generated by applying an alternating current (AC) peak-to-peak voltage of 4.5V to an electrode laminated onto the bottom of the flow chamber. The cell suspension traversed over the electrode region to obtain effective DEP. The target (cancer) cells were drawn to the electrode surface to allow collection at 15 μL/min through a port on the floor of the channel into a centrifuge tube (Fig 1C, step 2), while the non-target PBMC population was repelled upwards into a high velocity flow stream intended for the waste outlet. The ApoStream^®^ configuration and operating parameters including flow rate, AC amplitude and frequency, chamber geometry, and electrode geometry were empirically optimized to achieve enrichment of the target cancer cell populations.

### Spike-recovery experiments

Blood specimens (8 mL BD Vacutainer^®^ EDTA tubes [Becton Dickinson, Franklin Lakes, NJ]) for spiking experiments were drawn under informed consent from healthy donors at Frederick National Laboratory according to a protocol approved by the NCI-IRB. PBMC fractions were isolated using LeucoSep^®^ tubes (Greiner Bio-One, Monroe, NC) per the manufacturer’s instructions within 24 hours of the blood draw. PBMCs were re-suspended in ApoStream^®^ sample buffer at a concentration of 10^7^ PBMCs per mL before spiking with cancer cells. Multiple operators tested ApoStream^®^ instruments in both Frederick, MD (site 1) and Houston, TX (site 2) to account for user and instrument variability. Viable A549 cells, MDA-MB-231 cells, and ASPS-1 [7] cells pre-stained with CellTracker^®^ Green vital stain were spiked into Ficoll-isolated PBMCs at a concentration of either ~1000 or ~50 cancer cells per 1 mL buffer and 10^7^ PBMCs (to ensure clearance of the limit of detection), and processed through the ApoStream^®^ RUO device under cell-line specific, optimized operating conditions. A 20 μL aliquot was reserved from the pre- and post-separation samples, and PBMC percent depletion was determined by counting with a hemocytometer. In this study, the same ApoStream^®^ operating parameters were used for all three cell lines except for the operating frequency, which was adjusted to accommodate differences in cell capacitance, membrane morphology, and size.

Crossover frequency—the frequency at which the DEP force experienced by a cell transitions from a negative (repulsive) to a positive (attractive) force—is dependent on the conductivity and electric permittivity (i.e., dielectric constant) of the cell and suspending medium [5, 8]. At a specific eluate buffer conductivity (30 mS/m), various solid tumors have low mean crossover frequencies (30–50 kHz) compared to the crossover frequency of major peripheral blood cell types (90-175kHz) [8, 9]; this difference facilitates the isolation of CTCs from a complex mixture of cells. By selecting an optimal electrical frequency, the ApoStream^®^ system is able to exert positive (attractive) forces onto CTCs while simultaneously exerting negative (repulsive) forces onto non-target cells, thereby effecting separation of CTCs from PBMCs. Because separation frequency correlates with contamination by PBMCs of harvested cells, pre-validation studies were performed at both sites to establish the optimal separation frequencies for individual cell lines to achieve the desired enrichment performance. The empirically-determined separation frequencies used for optimal enrichment performance were 65 kHz for A549 cells, and 85 kHz for ASPS-1 and MDA-MB-231 cells. Three sample runs without spiked cancer cells (“zero” spike level) were run to assess cancer cell *carryover* between ApoStream^®^ runs. A flow chart summarizing the experimental design of the study is provided in S1 Fig.

Three aliquots of the enriched fraction were counted on a glass slide under a fluorescence microscope (Fig 1C, step 3), and residual PBMCs were counted using methylene blue dye on a hemocytometer. Recovery was calculated as the number of enriched cells divided by the total number of cells processed through the instrument. Similarly, PBMC fold reduction was analyzed by dividing pre-ApoStream^®^ PBMC counts (~10 million) by post-ApoStream^®^ counts. Statistical analysis was performed with Microsoft Excel 2010.

### Selection of purification scripts for ASPS-1 cells

ASPS-1 purification was tested by varying the applied frequency, specimen injection rate, and collection rate from the electrode chamber. In a response surface model (RSM) analysis, applied frequency and collection rate were determined to be the significant factors affecting purification and yield of cancer cells, therefore the injection rate was held constant. Sample collection rate was varied from 18 to 35 μL/min, and crossover frequency from 65 to 85 kHz. Two runs on different instruments were conducted for recovery of ASPS-1 cells spiked into buffer or PBMCs, and the corresponding decrease in PBMC counts was assessed (S2 Fig).

### Blood sampling and processing

Blood samples were collected into 4-mL CPT tubes (Becton Dickinson, Franklin Lakes, NJ) from 6 patients with metastatic ASPS enrolled in clinical trials conducted by the National Cancer Institute’s Developmental Therapeutics Clinic at the NIH Clinical Center, Bethesda, MD. Subsequently, the PBMC fraction was isolated according to the CPT package insert. Blood specimens (4–8 mL) from 15 additional patients with soft tissue sarcomas and 9 healthy donors were collected in K_3_EDTA tubes (Cat.# 366450, BD Biosciences, San Jose, CA). PBMCs were isolated by Ficoll density gradient separation using LeucoSep tubes (Greiner Bio-One, Monroe, NC), as per manufacturer’s instructions.

### CTC enrichment using ApoStream^®^ and determination of marker specificity in healthy donors

CTC enrichment was performed at the Frederick National Laboratory for Cancer Research within 24 hours of blood collection and stored at –80°C until processing. Each isolated PBMC layer was resuspended in sample buffer and processed under specific, pre-determined operating conditions. Clinical specimen workflow is shown in S3 Fig. For the determination of cancer marker specificity, PBMC preparations from Ficoll fractions were generated from 12 healthy donors. Typical yields were 2 x 10^6^ cells per mL blood, and 1 x 10^4^ cells were spotted per well on Marienfield slides. The slides were then stained and processed following the same procedure as for the patient specimens. The number of false positives were back-calculated based on actual cell counts.

### Identification of ASPS cancer cells by detection of ASPL-TFE3 fusion

**Fig 2:**
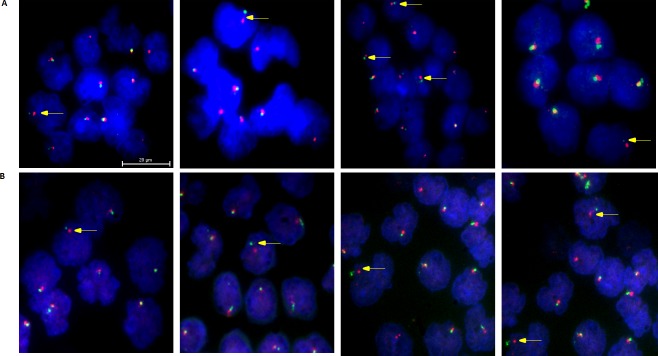
Representative images of CTCs from metastatic ASPS patients. **(A-B)** Specimens were probed with a two-color (green and red) probe set (for additional details, see Online Methods) and counterstained with DAPI. Co-localization of the probes (visualized as a yellow signal), indicates a normal Xp11.2 locus. Yellow arrowheads denote cells with *TFE3* rearrangements (separate green and red signals), which identify CTCs characterized by the separation of the probes. Scale bars indicate 20 μm.

The ApoStream^®^ settings determined empirically from the ASPS-1 cells purification experiments were applied for the recovery of CTCs from ASPS clinical samples; an applied frequency of 85 kHz and a collection rate of 25 μL/min were used on blood samples from patients with ASPS. The isolated cells were identified as ASPS cells by the presence of ASPL-TFE3 genetic rearrangement that serves to diagnose ASPS [10, 11]. Their identity was verified by TFE3 break-apart fluorescence in situ hybridization (FISH) (Fig 2), and the presence of the ASPL-TFE3 fusion protein [12] in those cells was confirmed by immunofluorescence detection (S4 Fig). Following enrichment with ApoStream^®^, cells were cytospun onto slides and fixed with Carnoy’s fixative for the FISH assay, or plated onto custom adhesion Marienfeld slides for immunophenotyping.

### Detection of sarcoma CTCs by immunofluorescence assay

Enriched cells were suspended in 200 μL PBS, plated onto Marienfeld^®^ adhesion slides (Azer Scientific, Morgantown, PA) and allowed to attach for 40 min at 37°C. Adhered cells were fixed with 10% neutral buffered formalin (NBF) (Sigma Aldrich, St. Louis, MO) for 10 min at room temperature. Fixed slides were stored at –80°C until further processing. Formalin fixed cells were subsequently permeabilized with ice-cold methanol for 5 min, and stained with a cocktail of antibodies against a panel of markers (details in S1 Table) for immunofluorescence. All antibodies were tested by Western blots (data not shown). The use of filter cubes on the microscope allowed for multiple phenotypic biomarkers to be included per detection channel, thus increasing throughput compared to spectral imaging methods which are limited by the number of available visible light channels. Nuclei were counterstained with 4',6-diamidino-2-phenylindole dihydrochloride (DAPI), and all slides were allowed to cure overnight at 25 ± 3°C in the dark before being stored at –20°C. See S5 Fig for validation of antibodies using control cells lines and matched isotypes.

### Validation of TLE1 as marker of sarcoma by western blot

Cell lysates from HT-1080, MES-SA, SK-LMS-1, SW684, SW872, SW982, ASPS-1, A375 cell lines (from ATCC; Manassas, VA) and PBMCs from study participant blood samples were prepared in cell extraction buffer (Invitrogen) containing protease inhibitor and PMSF, and their protein concentrations were determined using the BCA assay (Thermo Scientific, Waltham, MA). Fifty micrograms of protein were loaded per well for all cell lines. The proteins were separated on 4–12% precast gels (BioRad, Hercules, CA) by SDS-PAGE. The separated proteins were then blotted onto Nitrocellulose membranes. Membranes were blocked with blocking buffer (LI-COR Biosciences, Lincoln, NE) for 1 h at room temperature. The blots were probed with antibodies against TLE1 and GAPDH, overnight at 4°C. Corresponding secondary antibodies were purchased from LI-COR Biosciences (Lincoln, NE) and used at 1:10,000 dilution. Blots were scanned using Odyssey IR Imager (LI-COR Biosciences, Lincoln, NE).

## Results

### Cross-site spike-recovery validation

The mean cancer cell recovery, standard deviation, and CV were calculated for data collected at both sites (Table 1). Pass/fail acceptance criterion was defined prior to study initiation as a coefficient of variation (CV) < 20% for cancer cell recovery performance between sites. Validation results met acceptance criterion; all 3 cell lines at both spiking levels passed the validation studies, with average cancer cell recoveries at the 1000- and 50-cell spiking levels of ~68% and ~55%, respectively. At the 50-cell spiking level, the lower recovery percentage was expected due to inherent cell loss in the tubing, flow chamber, and pipette tips which make measurements at this spiking level more susceptible to measurement error (± 5 cells creates a 10% error).

**Table 1:** Summary of spiked cancer cell experiments at 2 sites.

Cell line and spike level	Spiked cancer cell recovery									
	Site 1			Site 2			Statistics			Validation
	Run 1	Run 2	Run 3	Run 1	Run 2	Run 3	Mean (%)	SD (%)	CV (%)	Pass/Fail
**A549**										
1000-cell	67.2	70.1	74.4	71.6	74.0	62.4	69.9	4.6	6.5	PASS
50-cell	62.2	69.8	60.0	50.0	51.0	42.0	55.8	10.0	17.9	PASS
**ASPS-1**										
1000-cell	68.0	69.4	66.7	71.5	59.6	54.5	64.9	6.5	10.1	PASS
50-cell	48.9	47.4	50.8	50.7	62.7	57.1	52.9	5.8	11.0	PASS
**MDA-MB-231**										
1000-cell	68.1	76.5	70.2	63.1	59.9	69.2	67.8	5.8	8.6	PASS
50-cell	52.7	60.3	44.2	50.0	60.1	64.2	55.3	7.6	13.7	PASS

Spiked cancer cell recovery at cancer cell spike levels 1000 and 50 per 10^7^ PBMCs after ApoStream^®^ separation at two testing sites. The PASS/FAIL acceptance criteria was defined prior to study initiation as coefficient of variation (CV) < 20% for cancer cell recovery performance between the two testing sites.

### Cross-site CTC enrichment

Enrichment of CTCs in the recovered fraction was measured by the fold reduction in PBMCs, calculated by dividing pre-ApoStream^®^ PBMC counts by post-ApoStream^®^ PBMC counts (S2 Table). As expected, the average PBMC fold reduction was much lower (315–851 fold) for ASPS-1 and MDA-MB-231 cells compared with A549 cells (1,821-3,414 fold) due to the higher operating frequency (85 kHz) required for optimal recovery of ASPS-1 and MDA-MB-231 cells, thus indicating overlapping distributions of the physical properties of those spiked cancer cell and PBMC populations (crossover frequency ~ 175 kHz). Of the 10^7^ PBMCs at input, the average absolute PBMC counts remaining in the single-pass purified samples were ~6,000 for A549 cells (65 kHz) and ~35,000 for ASPS-1 and MDA-MB-231 cells (85 kHz).

### Analysis of CTCs from patients with ASPS

Blood samples from 6 patients with ASPS were processed using the run conditions validated in the ASPS-1 spiking experiments described above (85 kHz crossover frequency and 25 μL/min sample collection flow rate). The enriched fraction was assessed by TFE3 break-apart FISH assay for the ASPL-TFE3 fusion. Representative images of these cell fractions are displayed in Fig 2, revealing that a sub-population of enriched cells carried the hallmark gene rearrangement. Between 53 and 168 CTCs positive for the rearrangement were observed per 1000 nucleated cells counted (mean 109; median 98) in the 6 patient specimens (Table 2). Analysis of ApoStream^®^ operating parameters captured by the instrumentation indicated very similar performance on ASPS patient specimens compared to ASPS-1-spiked PBMC specimen (S6 Fig).

**Table 2:** TFE3 break-apart FISH analysis of ASPS specimens.

Patient No.	Percent of nuclei positive for TFE3 break-apart FISH	Number of nuclei positive for TFE3 break-apart FISH per 1000 nucleated cells
1	10	103
2	9	93
3	17	168
4	5	53
5	9	88
6	15	148

Enumeration of cells with TFE3 break-apart FISH pattern in six patients with metastatic ASPS.

For comparative purposes, we also tested the detection of ASPS CTCs employing specific monoclonal antibodies against ASPL-TFE3 type 1 fusion protein [12] and vimentin (S7 Fig), as cell-surface vimentin (CSV) has been proposed as a marker of circulating sarcoma cells [13]. S3 Table summarizes the results from 4 specimens obtained from 3 patients. The overall detection rate was lower with this approach compared to FISH. Interestingly, in one patient ASPL-TFE3-T1^+^/VIM^-^ cells were also detected, further illustrating the phenotypic variability of circulating ASPS cells.

### Analysis of CTCs from patients with sarcomas

**Fig 3:**
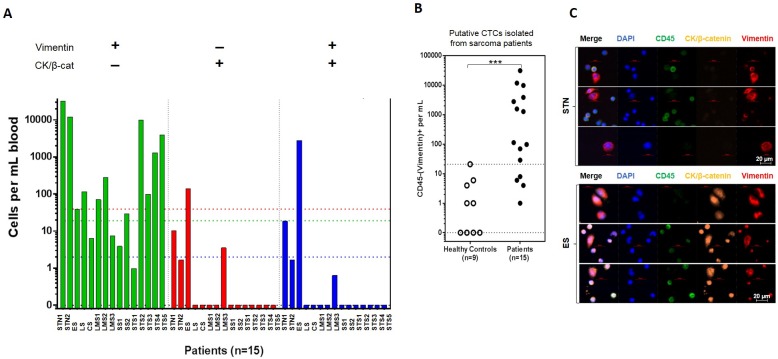
Enumeration and phenotypic characterization of CTCs from patients with sarcoma. Blood from advanced sarcoma patients and healthy donors were processed using the ApoStream^®^ device. Healthy donors (n = 9, open circles) were used to demonstrate assay specificity. **(A)** In 4 of the 15 sarcoma patients, phenotypic heterogeneity was seen. Colored dotted lines correspond to the assay cut-off for each of the phenotype measured. STN, soft tissue neoplasm; ES, embryonal cell sarcoma; LS, liposarcoma; CS, chondrosarcoma; LMS, leiomyosarcoma; SS, synovial sarcoma; STS, soft tissue sarcoma. **(B)** The advanced sarcoma patients (n = 15, closed circles) had significantly higher numbers of cells with CD45^-^/VIM^+^ phenotype (*p* = 0.0002), with 73% of patient samples above the 21 cells/mL cut-off. **(C)** Gallery of CTCs from a patient with soft tissue neoplasm (STN; top) and a patient with embryonal cell sarcoma (ES; bottom). Putative CTCs isolated using ApoStream^®^ were identified using antibodies against CK/β-catenin (orange), vimentin (red), and a nuclear stain (DAPI), while excluding leukocytes with marker CD45 (green). Scale bars correspond to 20 μm.

As a control, blood samples from a 12-healthy-donor panel were analyzed. Due to the low concentration of CTCs in the blood, we assessed the number of false positives that could be expected when tracking these markers (S5 Table). This analysis confirmed that the exclusion of CD45^+^ cells is critical for specific enumeration of cancer cells, and revealed that the major source of false positive cells in blood from healthy donors is the pan-cytokeratin marker. Specimens from 15 patients with soft tissue sarcomas other than ASPS were also processed on ApoStream^®^ at crossover frequency of 85 kHz, infusion rate of 35 μL/min, and effluent rate of 15 μL/min; CTCs were identified using a multiplex phenotyping assay containing a cocktail of antibodies to CTC biomarkers vimentin (VIM), cytokeratin (CK), and β-catenin (β-cat), while excluding CD45^+^ cells. Our aim was to establish a phenotyping panel capable of identifying CTCs from a range of different sarcomas isolated by ApoStream^®^. Patient diagnoses are listed in S4 Table. Eleven patients (73%) had a predominance of CTCs with CD45^-^/ (CK/β-cat)^-^/VIM^+^ phenotype 17 cells/ml (patient specimen range per mL: 29–31,418 cells; mean: 5,739 cells; median: 1,576 cells) as shown in Fig 3A. In contrast, 6 out of 9 healthy donors had ≤ 1 CD45^-^/VIM^+^ cells/mL (healthy controls mean 3.7 cells/mL ± 6.8; Fig 3B). False positive rates were typically on the order of 1 cell per 10^4^ cells (see S5 Table and Fig 3B). A large number of cells exhibiting alternative phenotypes CD45^-^/(CK/β-catenin)^+^/VIM^-^ and CD45^-^/(CK/β-catenin)^+^/VIM^+^ were seen in the other 4 patients; the patient with embryonal cell sarcoma (ES) had the highest levels of those cell sub-populations. Fig 3B show the distribution of the three different phenotypes identified in the CD45^-^ population across all patients. Interestingly, cells with phenotypes CD45^-^/(CK/ β-cat)^-^/VIM^+^ and CD45^-^/(CK/β-cat)^+^/VIM^+^ exhibit very distinct morphologies, as can be seen in the representative micrographs from a patient with soft tissue neoplasm (STN) and a patient with ES, respectively (Fig 3C).

**Fig 4:**
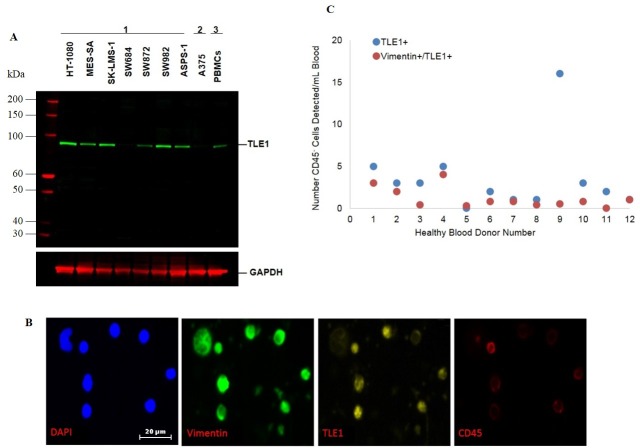
Validation of TLE1 as a tumor-specific marker of sarcoma. TLE1 (Transducin-Like Enhancer Protein 1) was identified as tumor-specific marker for sarcomas. **(A)** Western blot analysis of TLE1 (MW ~83 kDa) in: 1: sarcoma cell lines; 2: melanoma cell line; 3: PBMCs. GAPDH (MW ~37 kDa) served as a loading control. **(B)** CTCs isolated from the blood of a patient with sarcoma and characterized by TLE1 (yellow), vimentin (green), and DAPI (blue), while excluding CD45^+^ leukocytes (red) staining (4 channels); representative 80X images are shown. Scale bars indicate 20 μm. **(C)** Determination of false positive CD45-/Vimentin+/TLE1+ cells in a panel of healthy blood donors.

Because more cells than expected in one of the healthy donors matched the putative CTC phenotype CD45^-^/(CK/β-cat)^-^/VIM^+^, and in previous CTC work significant numbers of cells harvested did not express any of the cancer cell markers in our panel [14, 15], we sought to improve CTC identification by supplementing the detection expressed VIM with a biomarker specific to sarcomas. The transcriptional co-repressor TLE-1 (Transducin-Like Enhancer of Split 1) has been used previously as a marker of synovial sarcoma and other sarcomas of peripheral nerve sheath origin [16, 17], so we tested a purified sarcoma subset with an anti-TLE-1 antibody (Fig 4A and 4B; S8 Fig). In a second healthy donor panel of 12 specimens, cells that were CD45^-^/TLE1^+^ or CD45^-^/TLE1^+^/VIM^+^ were scored (Fig 4C). For 11 of 12 donors, the number of cells per milliliter of blood with either phenotype was low at ≤ 5; only one donor had an unexpectedly high number of CD45^-^/VIM^-^/TLE1^+^ cells with 16 per milliliter of blood. This discrepancy was eliminated by the addition of a second marker, vimentin. The median number of presumed false positive cells (± s.d.) detected, as defined by CD45^-^/VIM^+^/TLE1^+^ phenotype, was 0.8 ± 1.13 cells/mL (cutoff of 3 cells/ml; range 0–4 cells per mL of donor blood). Among the patients with liposarcoma, synovial cell or spindle cell sarcoma, the CD45^-^/VIM^+^/TLE1^+^ phenotyping panel identified CTCs in 8/9 patients, ranging from 18 to 3,711 cells (Table 3), consistent with the prior diagnosis of metastatic disease.

**Table 3:** Phenotypic characterization of putative CTCs with vimentin and TLE1.

Diagnosis	Blood Volume (mL)	Total CD45^-^ cell count per sample		
		TLE1^+^	TLE1^+^	Total TLE1^+^
		VIM^+^	VIM^-^	VIM^+/-^
Liposarcoma	5	35	0	35
Synovial Sarcoma	7	1,766	0	1,766
Synovial Sarcoma	4	3,711	4	3,715
Synovial Sarcoma	5	633	54	687
Mesothelioma (SCSS^a^)	4	4	0	4
Mesothelioma (SCSS^a^)	6	230	1	231
Mesothelioma (SCSS^a^)	7	85	27	112
Mesothelioma (SCSS^a^)	5	18	0	18
Mesothelioma (SCSS^a^)	7	67	0	67

Immunophenotyping of sarcoma cells isolated from the blood of 9 patients.

^a^SCSS: Spindle Cell Sarcomatoid subtype.

## Discussion

CTCs play an important role in metastasis as they are shed from primary tumors and tumor metastases into the vasculature [18, 19]. Efficient isolation and characterization of these cells is challenging as they are so rare—approximately 1 CTC per 10^9^ blood cells [20]. Furthermore, very few studies have reported isolation of CTCs from patients with non-epithelial cancers such as sarcomas [13], and the challenges associated with the detection of CTCs in sarcoma are widely recognized [21]. A variety of batch mode approaches have been proposed for the efficient isolation of CTCs from peripheral blood specimens, including negative selection enrichment [22–24], size filtration [25, 26] and dielectrophoresis (DEP) on chips [4, 27]. These methods exploit differences between biological and/or physical properties of CTCs and normal blood cells [5] rather than use antibodies to the epithelial marker EpCAM, which is not expressed by cells derived from sarcoma tumors. In the case of DEP field-flow assist, which is implemented in ApoStream^®^, differences in morphology, size, and total capacitance of the cell allow for discrimination between blood cells and CTCs [4, 28].

This work demonstrates comparable performance of ApoStream^®^ across two laboratories for 6 instruments and multiple operators at spike levels relevant to clinical specimens, and against normal PBMC backgrounds significantly higher than those observed in the advanced cancer patient population. The method was reproducible for 3 different human tumor cell lines with considerably different phenotypes. All data were reported from a single pass through the instrument; however, two passes have also been performed producing improved CTC purity (data not shown). The high standard deviation for PBMC fold reduction could be attributed to several sources: variability in donor blood samples, variability introduced by the cell counting method, and variations in pre-ApoStream^®^ total cell concentration and sample loading. Changes to the ApoStream^®^ processing protocol, including improved sample loading software script, have been implemented to reduce the variation in PBMC contamination were made subsequent to this study. In addition, other methods such as multi-pass processing can be implemented if further reduction in PBMC contamination is required. ApoStream^®^ has the flexibility in parameter settings to drive recovery or purity, depending on the downstream applications.

ASPS was selected for validation studies because its characteristic hallmark *TFE3* genetic rearrangement allows for unambiguous identification of tumor cells enriched from blood samples with the ApoStream^®^ device. We successfully employed the DEP operating parameters established for the ASPS-1 cell line in the cross-validation studies to isolate CTCs from blood samples of all 6 patients with metastatic ASPS, and confirmed their tumor origin using TFE3 break-apart FISH for the detection of the chromosomal translocation of ASPL and TFE3 genes [10]. This demonstration is of clinical relevance because although significant clinical progress has been made in treatment of the disease [29], ASPS is an extremely rare cancer of children and young adults with a poor prognosis, and for which the mechanism of metastasis is not well understood. Isolating CTCs with ApoStream^®^ will facilitate pharmacodynamic studies and patient monitoring that were precluded by the difficulty to obtain solid biopsies in this young patient population.

The FISH approach did not enable absolute cell counts. To facilitate future studies in which biomarker activity could be determined and exact cell counts obtained, we demonstrated that the ASPS cells isolated using the device could also be identified with antibodies generated to the fusion gene product. Immunophenotyping of ApoStream^®^-enriched samples from 3 of those patients with specific antibodies against ASPS type 1 (ASPL-TFE3-T1 translocation) and vimentin also identified ASPS CTCs, although with a lower detection rate (S3 Table). This is presumably due to the specificity of the monoclonal antibody for one of the two common rearrangement events, and due to the requirement for significant synthesis of the fusion protein for detection. The data presented in Fig 3 demonstrate unexpected phenotypic variability in the isolated CTCs. It has been reported that phenotypic variability in carcinomas—as assessed by analysis of circulating DNA, CTCs and solid tumor biopsies—is quite high, so that there is no “gold standard” [30]. As an alternative, analysis of small blood volumes (e.g. 2 mL samples) taken at different times of the day or on different days, and binning of the results may provide a dynamic clinical picture.

Having established that *bona fide* sarcoma cells could be isolated from the blood of ASPS patients with the ApoStream^®^ device, we next tested two panels of specimens from patients with varied sarcomas, using two different immunophenotyping approaches. In one approach, we used general markers of the mesenchymal phenotype and established a cut-off of the number of CD45^-^ cells in blood from healthy donors; then we scored blood specimens of patients with sarcoma, showing a greater number of putative sarcoma cells present in the patients compared to the healthy donors. Surprisingly, in the sarcoma patient population tested at baseline, we found evidence of a co-occurring epithelial phenotype in 4 of 15 patients, as evidenced by the presence of a CD45^-^/(CK/β-cat)^+^/VIM^-^ population. This revealed unexpected heterogeneity of CTC phenotypic profiles in patients with sarcoma. In a second approach, we looked for combined expression of the established sarcoma marker TLE1 with vimentin in CD45^-^ cells in another panel of sarcoma patients, demonstrating the ability to identify putative CTCs present in much higher numbers than the median number of presumptive false positive cells in the healthy donor population. These results highlight the necessity for multiplexing phenotypic markers to increase confidence of cell identification following isolation from the blood.

Given the recently reported passaging of CTCs in mice to establish tumors [31, 32], an important attribute of the DEP field-flow assist (4) approach is the ability to collect viable CTCs for further research purposes. The viability of cancer cells processed with ApoStream^®^ was already established [4]; the study presented here is an initial proof of the research- and clinical-readiness of the ApoStream^®^ instrument, which will facilitate the exploration of patient-derived models for uncommon cancers, including sarcomas, for cancer biology and pharmacological studies at NCI. Coupled with selection for biomarkers specific to sarcoma subtypes such as TLE1 or ASPL-TFE3, the ApoStream^®^ device will facilitate CTC isolation and analysis for anticancer drug development, longitudinal monitoring of pharmacodynamic drug effects, and disease management for patients with carcinoma or sarcoma alike.

## Supporting information

S1 Fig Flow chart of study experimental design. Flow chart summarizing the experimental design for the cross-site validation study.
(TIF)

S2 Fig Selection of ApoStream^®^ operating parameters for ASPS-1 cells. Percentage recovery of ASPS-1 cells and decrease in PBMC count using 3 purification scripts. Two runs on different instruments were performed for each script. The parameters in the legend are defined as: applied frequency, sample injection rate in μL/min, sample collection rate in μL/min.
(TIF)

S3 Fig Clinical specimen workflow. Procedure for the analysis of blood samples from patients with **(A)** ASPS and **(B)** other sarcomas.
(TIF)

S4 Fig Presence of type-1 ASPL-TFE3 genetic rearrangement. ASPL-TFE3 fusion in ASPS-1 cells demonstrated by **(A)** TFE3 break-apart FISH assay and **(B)** ASPL-TFE3 fusion IFA. ASPL-TFE3 type 1 and ASPL-TFE3 type 2 antibodies developed by Vistica D.T. et al. (12) were purchased from the Developmental Studies Hybridoma Bank (DSHB) at the University of Iowa as culture supernatants and used at 5 μg/mL concentration. Scale bar corresponds to 25 μm.
(TIF)

S5 Fig Validation of antibodies using control cell lines. Fluorophore-labeled antibodies against leukocyte marker CD45 (red), CK/EpCAM/β-cat (orange), tumor markers MUC1/CEA (green), and nuclear stain DAPI (blue) were evaluated in control cell lines Ls174T, HT-29, MDA-MB-231 (carcinomas), A375 (melanoma), and in human PBMCs. Scale bar corresponds to 25 μm.
(TIF)

S6 Fig DEP parameter profiles. Line graphs tracking the real-time change in key DEP parameters (in y-axis) conductivity (mS/m, blue line), frequency (kHz, pink line), voltage (V, green line) and current (A, red line) versus run time (in seconds) on the x-axis for **(A)** a representative ASPS clinical specimen and **(B)** ASPS-1 cells spiked into PBMCs. Note that the profile of the conductivity changes is exactly the same as the profile of the frequency changes due to the fact that the applied DEP frequency is directly proportional to the conductivity of the medium.
(TIF)

S7 Fig Identification of ASPS CTCs with vimentin and ASPL-TFE3 type 1 fusion protein. Representative images of ASPS cells isolated from a patient and labelled with DAPI nuclear stain, FITC, TRITC, and Cy5-conjugated monoclonal antibodies to vimentin, ASPL-TFE3 type 1 fusion protein, and CD45, respectively. The scale bars indicate 5 μm.
(TIF)

S8 Fig HT-1080 fibrosarcoma cells express vimentin and TLE1. Representative images of HT-1080 fibrosarcoma cells labelled with DAPI nuclear stain, FITC, TRITC, and Cy5-conjugated monoclonal antibodies to vimentin, TLE1, and CD45, respectively. The scale bars indicate 12 μm.
(TIF)

S1 Table Panel biomarkers. Different markers used in the study including reagent information and associated disease types.
(DOCX)

S2 Table PBMC fold reduction after ApoStream^®^ separation at two testing sites. (DOCX)

S3 Table Identification of circulating tumor cells from the blood of patient with ASPS. Circulating tumor cells were first purified with the ApoStream^®^ device. Phenotype characterization was performed using monoclonal antibodies specific to the ASPL-TFE3 type 1 fusion protein and to vimentin.
(DOCX)

S4 Table Diagnoses of 15 patients with soft tissue sarcomas. (DOCX)

S5 Table Enumeration of different phenotypes in 1 mL of blood from 12 healthy donors. (DOCX)
