# Supplementary figures and images for: Antibody-independent capture of circulating tumor cells of non-epithelial origin with the ApoStream® system
Source: PLoS One. 2017 Apr 12;12(4):e0175414. doi: 10.1371/journal.pone.0175414 (PMC5389826; doi:10.1371/journal.pone.0175414)

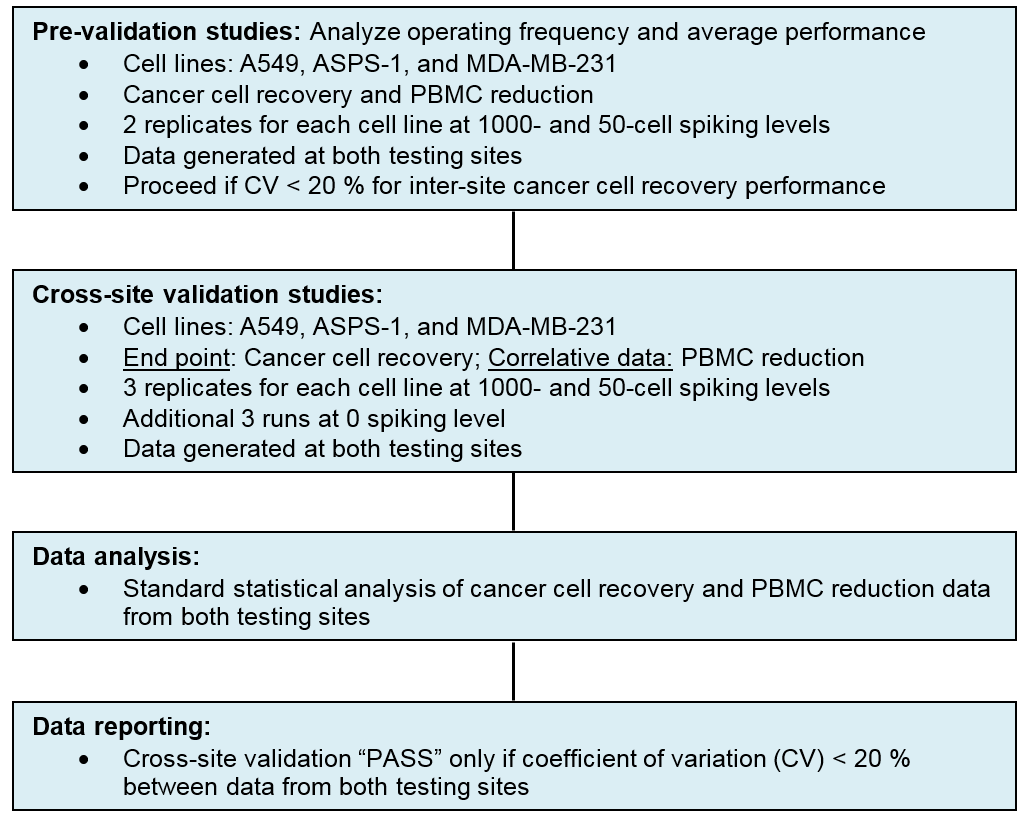

Supplement: S1 Fig — Flow chart summarizing the experimental design for the cross-site validation study. (TIF) [file pone.0175414.s001.tif]

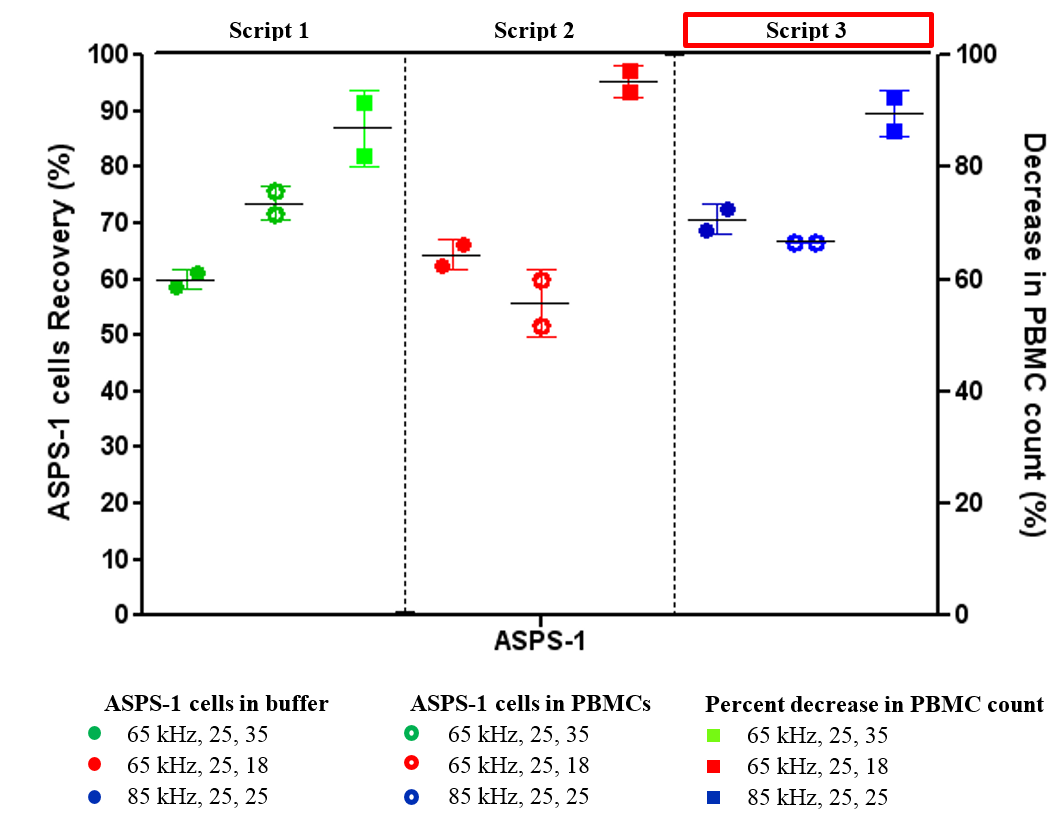

Supplement: S2 Fig — Percentage recovery of ASPS-1 cells and decrease in PBMC count using 3 purification scripts. Two runs on different instruments were performed for each script. The parameters in the legend are defined as: applied frequency, sample injection rate in μL/min, sample collection rate in μL/min. (TIF) [file pone.0175414.s002.tif]

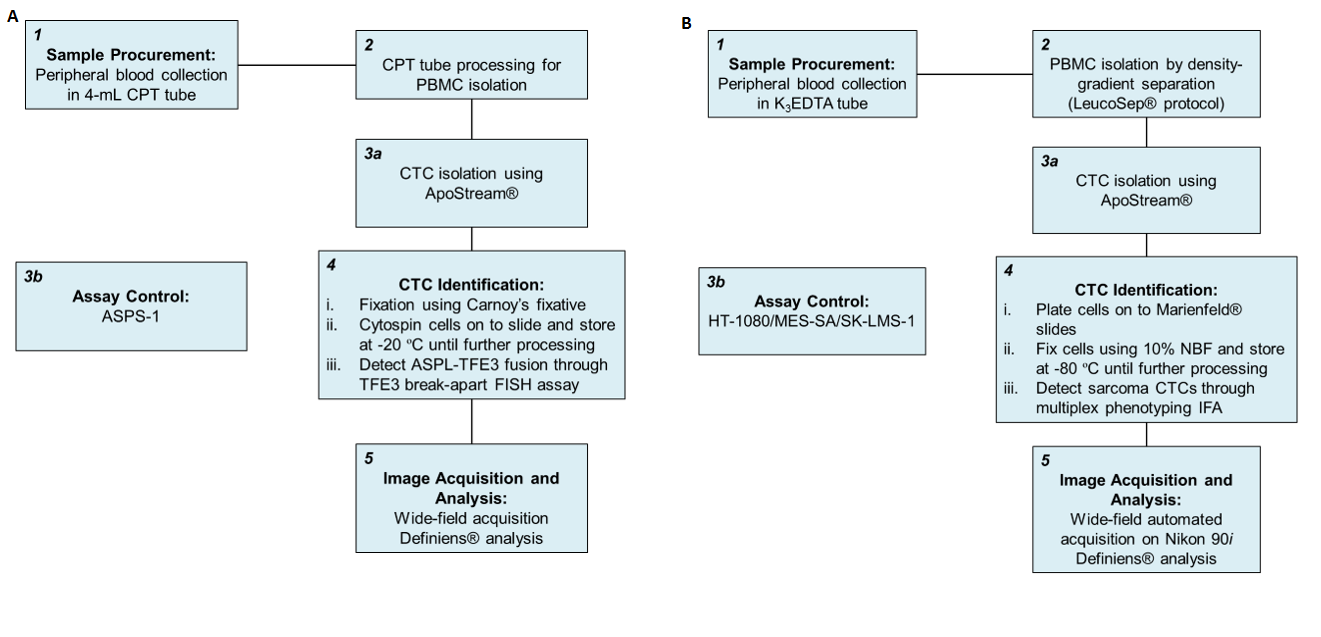

Supplement: S3 Fig — Procedure for the analysis of blood samples from patients with (A) ASPS and (B) other sarcomas. (TIF) [file pone.0175414.s003.tif]

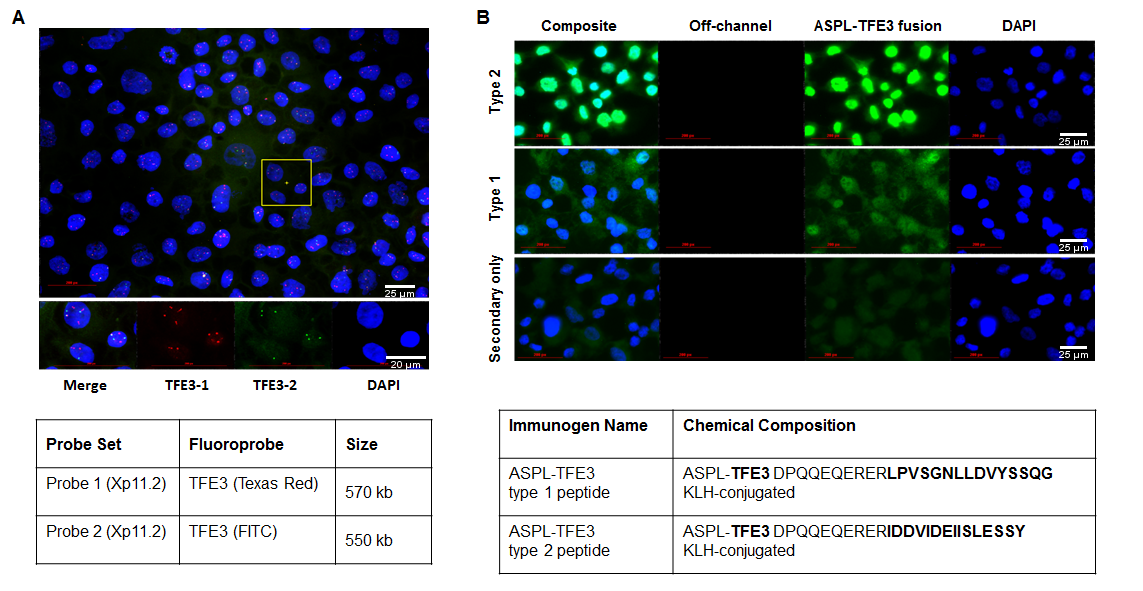

Supplement: S4 Fig — ASPL-TFE3 fusion in ASPS-1 cells demonstrated by (A) TFE3 break-apart FISH assay and (B) ASPL-TFE3 fusion IFA. ASPL-TFE3 type 1 and ASPL-TFE3 type 2 antibodies developed by Vistica D.T. et al. (12) were purchased from the Developmental Studies Hybridoma Bank (DSHB) at the University of Iowa as culture supernatants and used at 5 μg/mL concentration. Scale bar corresponds to 25 μm. (TIF) [file pone.0175414.s004.tif]

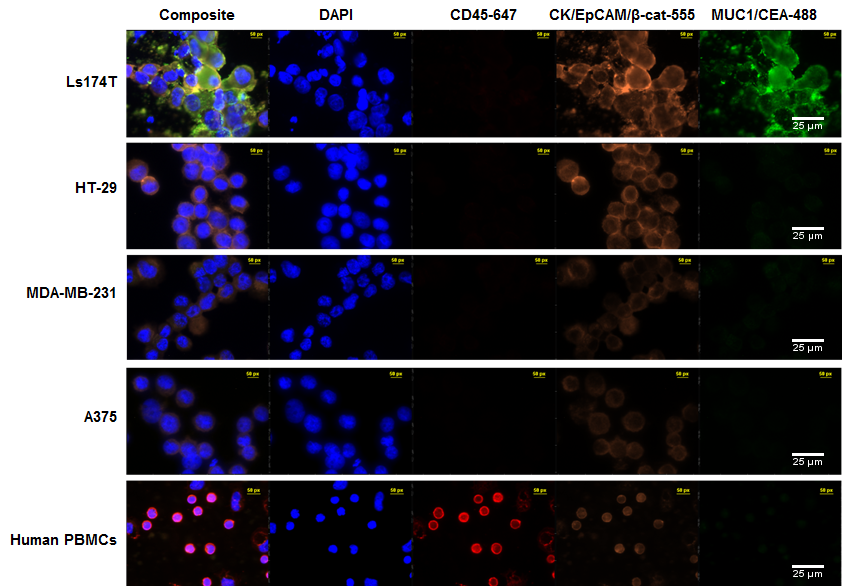

Supplement: S5 Fig — Fluorophore-labeled antibodies against leukocyte marker CD45 (red), CK/EpCAM/β-cat (orange), tumor markers MUC1/CEA (green), and nuclear stain DAPI (blue) were evaluated in control cell lines Ls174T, HT-29, MDA-MB-231 (carcinomas), A375 (melanoma), and in human PBMCs. Scale bar corresponds to 25 μm. (TIF) [file pone.0175414.s005.tif]

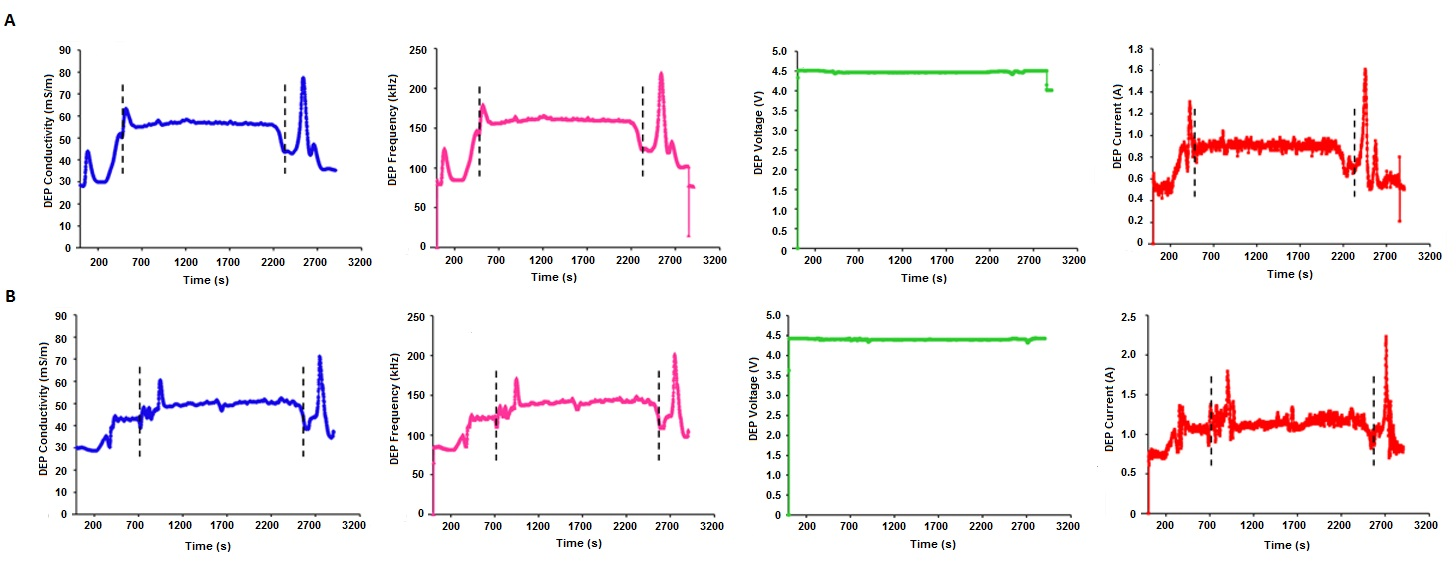

Supplement: S6 Fig — Line graphs tracking the real-time change in key DEP parameters (in y-axis) conductivity (mS/m, blue line), frequency (kHz, pink line), voltage (V, green line) and current (A, red line) versus run time (in seconds) on the x-axis for (A) a representative ASPS clinical specimen and (B) ASPS-1 cells spiked into PBMCs. Note that the profile of the conductivity changes is exactly the same as the profile of the frequency changes due to the fact that the applied DEP frequency is directly proportional to the conductivity of the medium. (TIF) [file pone.0175414.s006.tif]

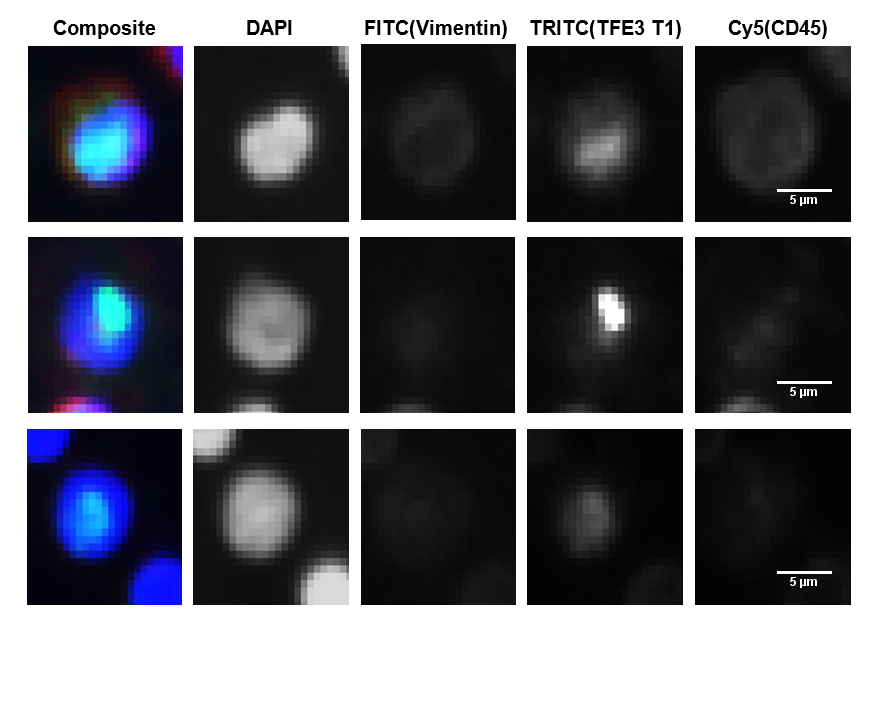

Supplement: S7 Fig — Representative images of ASPS cells isolated from a patient and labelled with DAPI nuclear stain, FITC, TRITC, and Cy5-conjugated monoclonal antibodies to vimentin, ASPL-TFE3 type 1 fusion protein, and CD45, respectively. The scale bars indicate 5 μm. (TIF) [file pone.0175414.s007.tif]

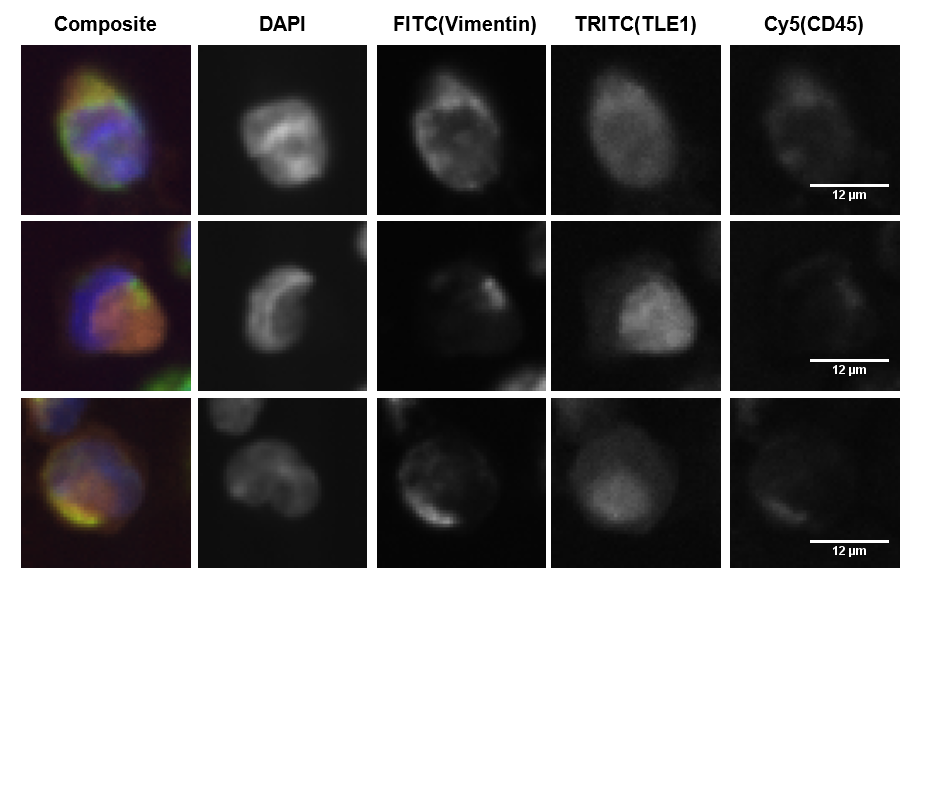

Supplement: S8 Fig — Representative images of HT-1080 fibrosarcoma cells labelled with DAPI nuclear stain, FITC, TRITC, and Cy5-conjugated monoclonal antibodies to vimentin, TLE1, and CD45, respectively. The scale bars indicate 12 μm. (TIF) [file pone.0175414.s008.tif]
