# Supplementary material for: Antibody-independent capture of circulating tumor cells of non-epithelial origin with the ApoStream® system
Source: PLoS One. 2017 Apr 12;12(4):e0175414. doi: 10.1371/journal.pone.0175414 (PMC5389826; doi:10.1371/journal.pone.0175414)
Supplement: S1 Table — Different markers used in the study including reagent information and associated disease types. (DOCX) [file pone.0175414.s009.docx]

**S1 Table. Panel biomarkers.**

| **Target** | **Panel** | **Disease type (Reference)** | **Phenotype** | **Clone**  **(Species, Isotype)** | **Vendor** | **Fluorochrome** |
| --- | --- | --- | --- | --- | --- | --- |
| **ASPL-TFE-3  type 1 fusion protein** | sarcoma | ASPS (10, 11, 12) | sarcoma | Ms, IgG1 | Developmental Hybridoma Studies Cell Bank, University of Iowa | indirect |
| **TLE1** | sarcoma | Synovial sarcoma  Spindle cell sarcoma  Liposarcoma (15, 16) |  | Rb, EPR9386(2) | AbCam | indirect |
| **Pan-cytokeratin (CK)^¥^** | Carcinoma/Sarcoma | - | Epithelial / Mesenchymal | C11  (Ms, IgG1) | CST | AlexaFluor®555 |
| **Epithelial cell adhesion molecule (EpCAM)^¥^** | Carcinoma |  |  | VU1D9  (Ms, IgG1) | CST | AlexaFluor®555 |
| **β-catenin (β-cat)^¥^** | Carcinoma/Sarcoma | - |  | E247  (Rb, IgG) | Epitomics | AlexaFluor®546 |
| **Vimentin** | Sarcoma | Sarcoma (20, 21) |  | V9  (Ms, IgG1) | SCBT | AlexaFluor®647 |
| **CD45** | Carcinoma/Sarcoma | Exclusion marker (N/A) | Hematopoietic | F10-89-4  (Ms, IgG_2a_) | AbD Serotec | AlexaFluor®647/488 |

Different markers used in the study including reagent information and associated disease types. Ms: mouse, Rb: rabbit; SCBT: Santa Cruz Biotechnology, CST: Cell Signaling Technology. ^¥^Refers to custom conjugated antibodies.
