# Supplementary material for: Antibody-independent capture of circulating tumor cells of non-epithelial origin with the ApoStream® system
Source: PLoS One. 2017 Apr 12;12(4):e0175414. doi: 10.1371/journal.pone.0175414 (PMC5389826; doi:10.1371/journal.pone.0175414)
Supplement: S2 Table — (DOCX) [file pone.0175414.s010.docx]

**S2 Table. PBMC fold reduction after ApoStream® separation at two testing sites.**

|  | PBMC fold reduction | | | | | | | |
| --- | --- | --- | --- | --- | --- | --- | --- | --- |
| Cell line and spike level | Site 1 | | | Site 2 | | | Statistics | |
|  | Sample 1 | Sample 2 | Sample 3 | Sample 1 | Sample 2 | Sample 3 | Mean | SD |
| ***A549*** | | | | | | | | |
| 1000-cell | 844 | 3,530 | 2,314 | 1,356 | 537 | 2,344 | 1,821 | 1,119 |
| 50-cell | 5,488 | 3,125 | 1,957 | 2,911 | 6,986 | 18 | 3,414 | 2,492 |
| ***ASPS-1*** | | | | | | | | |
| 1000-cell | 426 | 741 | 731 | 193 | 323 | 76 | 415 | 275 |
| 50-cell | 323 | 469 | 576 | 130 | 214 | 176 | 315 | 176 |
| ***MDA-MB-231*** | | | | | | | | |
| 1000-cell | 666 | 353 | 232 | 385 | 287 | 772 | 449 | 218 |
| 50-cell | 216 | 331 | 238 | 2,140 | 1,829 | 349 | 851 | 885 |
