# Supplementary material for: Antibody-independent capture of circulating tumor cells of non-epithelial origin with the ApoStream® system
Source: PLoS One. 2017 Apr 12;12(4):e0175414. doi: 10.1371/journal.pone.0175414 (PMC5389826; doi:10.1371/journal.pone.0175414)
Supplement: S3 Table — Circulating tumor cells were first purified with the ApoStream® device. Phenotype characterization was performed using monoclonal antibodies specific to the ASPL-TFE3 type 1 fusion protein and to vimentin. (DOCX) [file pone.0175414.s011.docx]

**S3 Table. Identification of circulating tumor cells from the blood of patient with ASPS.** Circulating tumor cells were first purified with the ApoStream® device. Phenotype characterization was performed using monoclonal antibodies specific to the ASPL-TFE3 type 1 fusion protein and to vimentin.

|  | Image Channel | | |
| --- | --- | --- | --- |
| Specimen | L2  ASPL-TFE3-T1^–^/VIM^+^ | L3  ASPL-TFE3-T1^+^/VIM^–^ | L2+L3  VIM^+^/ASPL-TFE3-T1^+^ |
| Patient 7 (on therapy) | 960 | 11 | 9 |
| Patient 8 (pre-dose) | 4,253 | 0 | 7 |
| Patient 8 (on therapy) | 8,979 | 0 | 11 |
| Patient 9 (pre-dose) | 9,071 | 0 | 3 |
