# Supplementary material for: Antibody-independent capture of circulating tumor cells of non-epithelial origin with the ApoStream® system
Source: PLoS One. 2017 Apr 12;12(4):e0175414. doi: 10.1371/journal.pone.0175414 (PMC5389826; doi:10.1371/journal.pone.0175414)
Supplement: S4 Table — (DOCX) [file pone.0175414.s012.docx]

**S4 Table. Diagnoses of 15 patients with soft tissue sarcomas.**

| Type of Sarcoma | Patient Numbers |
| --- | --- |
| Soft tissue neoplasm (STN), NOS | 1, 2 |
| Embryonal cell sarcoma (ES) | 3 |
| Liposarcoma (LS) | 4 |
| Chondrosarcoma (CS) | 5 |
| Leiomyosarcoma (LMS) | 6, 7, 8 |
| Synovial sarcoma (SS) | 9, 10 |
| Soft tissue sarcoma (STS), non-rhabdomyosarcoma | 11, 12, 13, 14, 15 |

NOS: Not Otherwise Specified.
