# Supplementary material for: Antibody-independent capture of circulating tumor cells of non-epithelial origin with the ApoStream® system
Source: PLoS One. 2017 Apr 12;12(4):e0175414. doi: 10.1371/journal.pone.0175414 (PMC5389826; doi:10.1371/journal.pone.0175414)
Supplement: S5 Table — (DOCX) [file pone.0175414.s013.docx]

**S5 Table.**  **Enumeration of different phenotypes in 1 mL of blood from 12 healthy donors.**

| Healthy donor no. | CK^+^  CD45^-^ | CK^+^  CD45^+^ | MUC1/CEA^+^  CD45^-^ | MUC1/CEA^+^/CK^+^  CD45^-^ | MUC1/CEA^+^/CK^+^  CD45^+^ |
| --- | --- | --- | --- | --- | --- |
| 1 | 0 | 0 | 0 | 18 | 18 |
| 2 | 97 | 2,527 | 0 | 34 | 178 |
| 3 | 0 | 28 | 28 | 0 | 42 |
| 4 | 131 | 2,450 | 0 | 0 | 116 |
| 5 | 38.5 | 0 | 0 | 77 | 0 |
| 6 | 101 | 524 | 0 | 0 | 101 |
| 7 | 42 | 693 | 0 | 0 | 20 |
| 8 | 52 | 816 | 26 | 26 | 65 |
| 9 | 0 | 194 | 28 | 0 | 98 |
| 10 | 252 | 3,537 | 0 | 66 | 109 |
| 11 | 131 | 1,832 | 78 | 53 | 130 |
| 12 | 14 | 27 | 0 | 0 | 0 |
